# Off-Road Detection Analysis for Autonomous Ground Vehicles: A Review

**DOI:** 10.3390/s22218463

**Published:** 2022-11-03

**Authors:** Fahmida Islam, M M Nabi, John E. Ball

**Affiliations:** Department of Electrical and Computer Engineering, Mississippi State University, Starkville, MS 39762, USA

**Keywords:** autonomous ground vehicles, off-road environment, drivable ground, positive obstacles, negative obstacles

## Abstract

When it comes to some essential abilities of autonomous ground vehicles (AGV), detection is one of them. In order to safely navigate through any known or unknown environment, AGV must be able to detect important elements on the path. Detection is applicable both on-road and off-road, but they are much different in each environment. The key elements of any environment that AGV must identify are the drivable pathway and whether there are any obstacles around it. Many works have been published focusing on different detection components in various ways. In this paper, a survey of the most recent advancements in AGV detection methods that are intended specifically for the off-road environment has been presented. For this, we divided the literature into three major groups: drivable ground and positive and negative obstacles. Each detection portion has been further divided into multiple categories based on the technology used, for example, single sensor-based, multiple sensor-based, and how the data has been analyzed. Furthermore, it has added critical findings in detection technology, challenges associated with detection and off-road environment, and possible future directions. Authors believe this work will help the reader in finding literature who are doing similar works.

## 1. Introduction

For years, industry and academia have been interested in developing autonomous technologies, such as driverless vehicles. Driverless vehicles are also known as autonomous ground vehicles (AGV) [1], unmanned ground vehicles (UGV) [2], autonomous guided vehicles [3], or autonomous land vehicles (ALV) [4]. These terms refer to vehicles that can navigate without or with minimal human assistance [5]. They are one of the first modern applications in robotics research. From the early days, AGV has been constantly being improvised to provide advanced driver assistance, road safety, and collision avoidance [6,7,8]. However, research in the unstructured environment still falls behind compared to structured environments. Many uncertain factors are responsible for this, like lack of labeled dataset, accessibility of data, lack of applicable data [9], etc.

**Figure 1:**
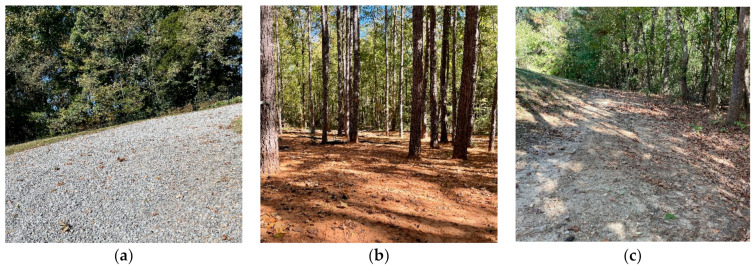
Examples of some off-road environments with unstructured roadways; The figure presents (**a**) rocky, (**b**) muddy, and (**c**) sandy road environments. Images are collected from the autonomous testing ground of the Center for Advanced Vehicular Systems (CAVS) at Mississippi State University (MSU).

Off-road environments are regions of suburban or non-urban, non-structured, or weakly structured road areas that lack well-defined routes and driving instructions like road signs, traffic signals, etc. Some good examples of off-road environments are forests, country roads, muddy or sandy roads, or terrain covered by emergent plants [10]. Oliver et al. [11] defined unstructured environments as situations or environments that “have not been previously adjusted to make it easier for a robot to complete a task”. In layman’s terms, an off-road environment can be any environment that does not have basic driving facilities, road instructions, and more challenging than usual conditions. Figure 1 shows some examples of off-road environments that have unstructured roadways and without proper driving facilities. Figure 1a–c shows rocky, muddy, and sandy road environments, respectively. 

The on-road environment is an urban area with structured roadways, along with necessary driving instructions, e.g., road signs, street markings, etc. On-road detection techniques involve lanes, traffic signals, road signs, pedestrians, vehicles, and building detection. Most of the objects on-road are specific and identifiable with a vision-based method. However, in off-road scenarios, there may not have any specific element. Therefore, it is very difficult to sense the environment. In the off-road environment, two major detections are essential to navigate smoothly. At first, it needs to identify the traversable or drivable ground where nothing can block the movement of the vehicle. Secondly, the vehicle needs to look for obstacles to find the appropriate path. Any prior knowledge about the environment facilitates the vehicle system to reach its goal at the minimum cost with the shortest distance.

Our fundamental goal is to present a brief overview of the primary methods of detection applied in off-road environments. In this paper, we examine the state-of-the-art detection techniques for AGV in the off-road environment. We have discussed the types of detection that need to be considered by the vehicles in the off-roads and the sensors or the methods used for those detections.

We have gathered and organized past works in a systematic way in which the problem of detection can be identified. The authors do not claim to provide all solutions recorded in the literature because doing so would be somewhat impossible. We hope that scholars working on this area will get a clear picture of the most relevant methodologies currently accessible in the literature by publishing this review.

The rest of this paper is structured as follows: Section 2 will provide a few related works, the novelty of this work, and classifications. Section 3, Section 4 and Section 5 will detail each category and the detection methods adopted by different works in the literature. Section 6 contains a comprehensive discussion, including key findings, major challenges, and future possibilities. Finally, Section 7 concludes the paper.

## 2. Related Works, Novelty, and Classifications

Many reviews and surveys are available in the literature covering the mobility of AGV or mobile robots in both on and off-road environments. We are providing the works done in off-road environments, as our research focuses on that.

The first review is presented by Chhaniyara et al. describing various terrain classification methods based on the mechanical property of the terrains that had been applied in previous planetary research in terrain trafficability analysis [12]. It is important to know about the geometrical properties of its terrain surface and surroundings for planetary rovers. Their approach differs from traditional terrain identification methods, focusing on remote and in situ sensing for terrain classification.

Conversely, Papadakis [13] provides a similar analysis but gathers different methods for analyzing terrain traversability based on sensory data. This paper classifies the methodologies into three broad categories-proprioceptive, geometric, and appearance-based. Hybrid approaches are also discussed with a brief overview of each category.

Few works highlight sensory information. A work presented by Ilas [14] summarizes the major sensor technologies used by AGV, along with their scope of the evaluation. AGV needs to observe its surrounding environment and make real-time decisions, requiring advanced sensing. This work provides the sensor information used in different types of environments in different vehicle prototypes and evaluates sensor development.

Babak et al. [15] reviewed the advancements in AGV technology, following brief history and the sensor technologies utilized in AGV. They briefly explained the recent sensor fusion techniques, advances in embedded software approaches, and the logic between sensory data and actuation decisions.

The work by Lynch et al. [16] focused on the sensors needed for guiding AGV’s navigation. They presented a brief overview of the sensors used for AGV navigation. In addition, a comparison of different sensors with their sensing capabilities, cost, and efficiency has been provided to guide the researchers.

Hu et al. [17] presented a survey on obstacle detection using sensor fusion in an off-road environment. A single sensor may have many limitations and cannot fully satisfy the requirements of obstacle detection, and multiple sensors can overcome this challenge. The authors have briefly described the state-of-the-art fusion techniques and suggested selecting the sensors based on the performance and the environment.

Guastella et al. [18] provided an incredible work, discussing the recent works highlighting learning-based methods to resolve environment perception. Perception gives the vehicle the information it needs to understand its own situation and its surroundings. They classified the learning-based method into two broad categories- terrain traversability and end-to-end method. Terrain traversability is further divided into classification, regression, and mixed of both methods. They mainly want to deal with autonomous mobility in the off-road environment.

Table 1 summarizes the review works presented in this section. These are the existing review works published. In the table, we included when the works were published, what areas had been covered, and what their work focused on. As shown, the existing literature focused either on remote sensing, sensor technology, or learning-based methods. We have also provided what we have presented in this work, how our work can contribute and how it differs from the existing literature. This paper includes the related outcomes for detection regardless of the detection method.

### 2.1. The Novelty of This Work

As mentioned earlier, this work aims to present a high-level overview of the methods adopted by researchers for detection techniques, specifically done in off-road environments. According to the previous work, several attempts have been made to focus on the off-road environment, traversability, sensing technologies, obstacle detection, and learning methods. However, no such effort has yet been made, considering all the factors of detection. In our work, we have not confined the detection to any specific object or technique; instead, we believe detection has a broad category.

This work covers different elements of detection possibly found in the off-road environment. We have considered all objects that need to be detected for smooth navigation. Sensor-based techniques, including single and multiple sensors, and analyzing algorithms, including machine learning, have been considered for the detection methods. This work has not included the techniques applied in the on-road or urban environment. Furthermore, this work has not looked for other factors that affect traversability, like path planning algorithms, perception problems, and positioning systems. More specifically, our study has not included surface roughness, slope, soil moisture, plasticity, and local position. 

### 2.2. Classification

**Figure 2:**
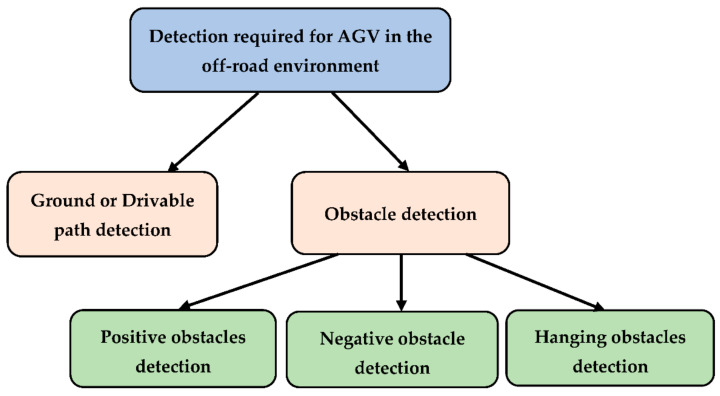
Classification of detection for AGV navigation in the off-road environment.

As we mentioned, we consider the detection analysis a broad study; therefore, grouping the same detection element helps readers connect similar works. In this section, we have classified the literature into multiple segments based on the detection factor. Figure 2 shows the categorization of detection for traversing AGV in the off-road environment. As shown in the figure, two major types can be identified within the scopes described in the previous section of AGV for detection in the off-road environment- drivable ground and obstacles.

**Figure 3:**
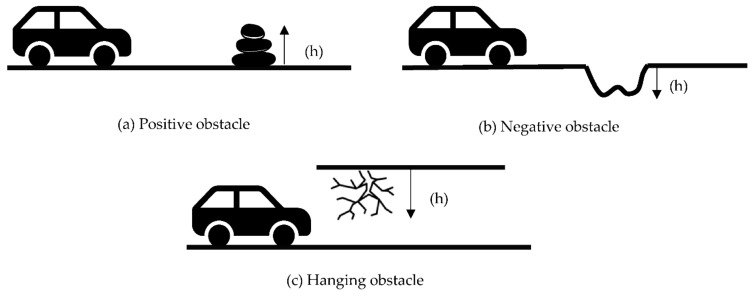
Different types of obstacles found in the off-road environment.

The obstacles have been further divided into three categories-positive, negative, and hanging obstacles. Figure 3 shows the different obstacles available in the off-road environment. We have found multiple papers for each major detection category. Each section has been further subdivided based on its detection method. However, we could not find any work that has been done solely for hanging obstacle detection in the literature. Therefore, we have not included the hanging obstacles in this study; however, it is an important aspect of the off-road environment. So, we have presented the hanging obstacle detection status as a future possibility at the end of this work.

The sections and subsections of this work have been presented below:

1. Ground or drivable pathway detection
  1.1 Single Sensor-Based Detection
  1.2 Multiple Sensor-Based Detection
2. Obstacle detection
  2.1 Positive obstacle detection
    2.1.1 Single sensor-based detection
    2.1.2 Multiple sensor-based detections
  2.2 Negative obstacle detection
    2.2.1 Missing data analysis
    2.2.2 Vision-based detection
    2.2.3 Other methods

**Figure 4:**
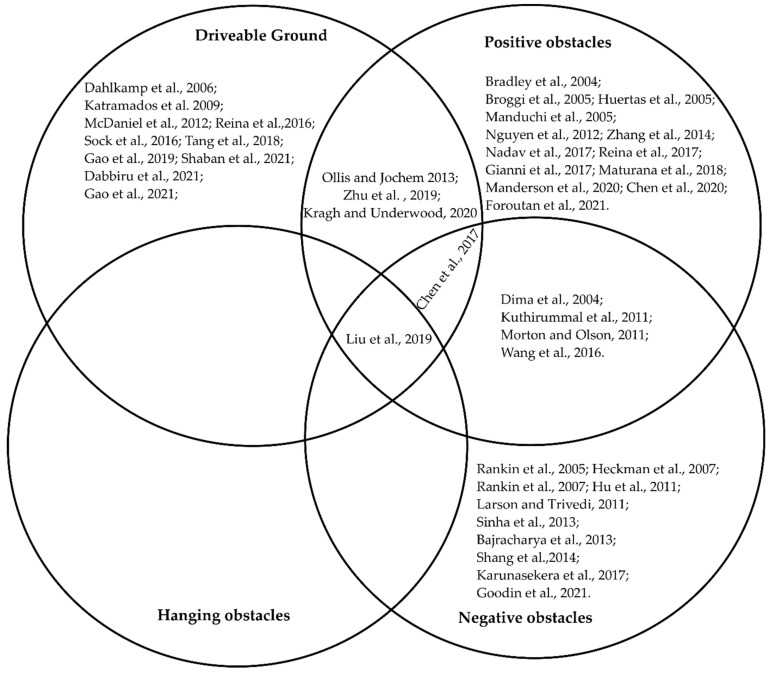
Distribution of the literature in different categories.

In Figure 4 we have demonstrated how the different detection works fit into each category using a Venn diagram representation. Readers will clearly visualize how much work has been published in each category. We have also found some works in the literature that can fit into multiple categories at the same time. The Venn diagram clearly shows those works where they fit. Therefore, it will be easy to understand which ones covered more than one detection. To avoid repetition, we have included the paper in that group based on the detection that has been emphasized.

## 3. Ground or Drivable Pathway Detection

One of the most important criteria in autonomous driving is identifying drivable regions. On-road pathways are easily identifiable because of their structure, color, smoothness, and horizontality. Off-road areas are more challenging, requiring advanced sensing equipment and techniques because, in off-road areas, roads are not the same in all areas. Instead, certain areas of the ground may be unsmooth, sloppy, and bumpy. In addition, having dense vegetation, grass, sand, or dirt, the driving pathway may not be distinguishable by a visual identifier. As a result, the AGV may lose its autonomous navigation capability.

### 3.1. Single Sensor-Based Detection

Liu et al. [19] used only 3D lidar to detect drivable ground, positive, negative, and hanging obstacles. They primarily detected obstacles to uncovering the traversable region in their work. They used 3D lidar points and analyzed the radial and transverse features. These features detect the obstacles, and then the leftover areas are defined as drivable regions for traversing AGV.

Gao et al. [20] also used lidar and proposed a deep-learning approach where they used the vehicle trails as input and obstacle marks as the label for the network. The suggested network topology was created considering the obscure and uncertain zone in the off-road environment. For their network, no human involvement is required to label the obstacles as it is done automatically. The main advantage of this network is that it takes data without labeling or weakly labeling but still provides a satisfactory result. 

Chen et al. [21] detected traversable road sections and obstacles in one unified model. They collected lidar image data and converted it to a lidar point cloud. A histogram map has been generated with the lidar point cloud, where the traversable road area can be visible in front of the vehicle. Apart from the traversable path, the obstacles are also visible around it. 

Katramados et al. [22] created a “traversability map” by extracting color and texture from images. For that, they collected camera data, mounted it on the top of the vehicle, and removed some unnecessary information. Then, they generated the map and adjusted the image’s temporal filtering, which helped to detect edges from blurry images. They removed lighting effects like shadows and reflections from the dataset, making the final detection result accurate. 

Shaban et al. [23] developed a deep learning model named Bird’s Eye View Network (BEVNet). This model took aerial images from the lidar sensor and semantically segmented them into four terrain classes- “free, low-cost, medium-cost, and obstacle”. The advantage of this model is that it can fill any gap with information using previous knowledge, and thus, it can overcome the problem of missing values for those areas where no lidar hits are found.

In another work, Gao et al. [24] suggested an approach based on contrastive learning using camera images. In contrastive learning, a single feature is trained for classification. They used a set of human-labeled bounding boxes as features and detected different traversable areas for this work. Those areas are semantically segmented to generate an understandable map of the environment. 

Overall, lidar and camera sensors have primarily been used for traversable ground detection when using only a single sensor. Furthermore, the deep learning method is quite interesting to the researchers, as they can overcome some limitations like uncertain zone, missing data, etc. 

### 3.2. Multi-Sensor-Based Detection

Zhu et al. [25] combined three lidars to detect three different types of obstacles and traversable paths. They used lidar odometry, which converts the detection output into a structured form. They recorded several findings, combining each with the following result. This combination uses the Bayesian theory, which provides a reward. However, this method will not work for long-distance traversability.

A drivable ground detection method in a dynamic environment has been presented by Dahlkamp et al. [26]. This method has been applied to the vehicle that participated and won in the “DARPA Grand Challenge robot race”. DARPA is the “defense advanced research projects agency”. A “drivability map” is created by a laser range finder and camera sensor with an appearance model. This vehicle was able to navigate through the desert terrain very fast. 

Mei et al. [27] designed an algorithm to detect the traversable area in the off-road environment. They captured images with a monocular camera. The image of the same area is also captured by a lidar-based and observed by a human. The far-field capability is also measured. The final traversable region is defined by comparing the image data with the three measurements. 

An unsupervised learning-based method has been developed by Tang et al. [28] for segmenting passable areas in unstructured environments. They used a deep convolutional neural network to classify free, obstacle, and unknown road areas. They used both camera and laser sensors for training data and generated automatic labeling. For testing data, only a monocular camera is sufficient.

Semantic segmentation, also known as image segmentation, is the process of allocating one of *N* predefined classes to every pixel of an image [29]. It is a deep learning algorithm that depends on a large set of labeled datasets. Dabbiru et al. [9] published a labeled dataset using semantic segmentation of three different vehicle types for an off-road environment. They used two 2D cameras and a 3D lidar sensor for data collection and annotated them based on the vehicle class.

Reina et al. [30] detected drivable ground by combining lidar and stereo data and two classifiers. Each classifier takes data from each sensor, and then the classification result is fused to get the final result. Likewise, Sock et al. [31] used lidar and camera data to measure road boundaries and shape. They generated two probabilistic maps with two different sensors and then classified the traversable region with a linear support vector machine (SVM). The two classification results have been fused with the Bayes rule. McDaniel et al. [32] proposed a method for detecting tree stems using an SVM classifier. They used a lightweight lidar scanner from a single point of view. This method has two steps where the non-ground points have been filtered out in the first step, and then SVM classifies the points that belong to the ground from the remaining.

In summary, fusing multiple sensors can provide a better detection result for drivable ground. Lidar and camera fusion is the most common fusion method. The classification result from two different sensors is being compared with the Bayesian rule. However, using multiple sensors may be costly. Table 2 encapsulates the works presented in Section 3. To keep this table simple and easily understandable, we have included the type of sensors and techniques used by each literature and what they detected.

## 4. Positive Obstacles Detection and Analysis

AGV considers any particle as an obstacle that obstructs its smooth driving. Navigating securely without colliding with objects or falling into gaps is a key criterion for AGV. Obstacle detection and navigation in an unknown environment are one of the major capabilities of AGV. Obstacles are multiple objects that hinder the usual speed of the vehicles or make it a complete stop. Dima et al. [33] defined obstacles as “a region that cannot or should not be traversed by the vehicle”. Pedestrians, vehicles, houses, trees, animals, boulders, giant cracks, vast quantities of water, snow, etc., can be considered obstacles. 

### 4.1. Single Sensor Based 

Huertas et al. [34], determined the diameters of trees from the stereo camera image to assess whether they constitute traversable obstacles using edge contours. Edge contours are generated from the stereo pair (left and right) images, which is called 3D fragment information. Then, this information is coded with orientation to confine processing to object borders to match the edges on opposing tree trunk boundaries. 

Maturana et al. [35] aim to differentiate small objects from large obstacles. They built a 2.5D grid map where they labeled terrain elevation, trail, and grass information. A 2.5D grid map is an image type where the height and depth information is provided. That information has been collected through lidar and other image data. The semantic map dataset is further trained and tested with a customized CNN for path planning and cost calculation.

Manderson et al. [36] presented a reinforcement learning method using labeled images. They collected images from the front end and overhead with various obstacles like vegetation, different rock kinds, and sandy paths. These images are used as inputs, and the labeling process is self-supervised. Value Prediction Networks (VPN) [37] have been used as a network. VPN is a hybrid network consisting of model-based and model-free architecture.

Nadav and Katz [37] presented a system with low computational cost. They used a smartphone to collect images, and they converted the images into a 3D point cloud model and processed it to detect obstacles and distance information. Another benefit of this system is that it can operate individually without other sensors.

Broggi et al. [38] presented a system that provides real-time obstacle detection using “stereoscopic images”. These images are collected from a moving vehicle through two cameras on the left and right sides. Then, the system calculates “V-disparity”, which determines the pitch oscillation of the camera from vehicle movement. V-disparity is a method that uses a single pair of stereo pictures to determine the camera’s pitch angle at the moment of acquisition [39]. The obstacles are then identified and mapped in real-world coordinates.

Foroutan et al. [40] presented a different approach than typical obstacle detection. They look into the effect of understory vegetation density on obstacle detection and use a machine learning-based framework. For that, they take point cloud data from the lidar sensor using an autonomous driving simulator. If the understory vegetation increases, the classification performance decreases. 

A laser-based system with the Sober algorithm and the Gaussian kernel function has been applied by Chen et al. [41]. The goal is to group each obstacle’s point clouds, so the super-voxel has been optimized with the Euclidean clustering technique. Then, “the Levenberg–Marquardt back-propagation (LM-BP) neural network” has been applied to extract the features of the obstacles.

Zhang et al. [42] provided a faster detection method using stereo images. The detection method has two stages. In the first stage, it rapidly identifies the easily visible obstacles, and then in the second stage, it uses space-variant resolution (SVR) [42] to improve small obstacle detection. SVR is an algorithm that analyzes the geometric features and the level of interest in each area. However, SVR has a very high computation cost. 

Overall, cameras are widely used sensors for obstacle detection. However, lidar and laser also provide good detection. While identifying the obstacles from the image, different image processing techniques and detection algorithms have been applied in different works. Some took different approaches by considering small objects. 

### 4.2. Multi-Sensor Based

Kuthirummal et al. [43] presented an approach that can be applied to lidar and camera sensors. They created a grid-based obstacle map to define traversability. To map the obstacles in each cell, they calculate the elevation histogram and plot them on the graph with the label. Thus, the information about obstacles can be known.

Manduchi et al. [44] presented a sensor processing algorithm using two sensors. A color stereo camera is used for detecting obstacles based on color and terrain type. A single-axis ladar classifies the traversable and large obstacles. The camera is mounted on the vehicle’s top, while the ladar is placed in the lower portion. These two systems provide better navigation together.

Reina et al. [45] built perception algorithms to improve the autonomous terrain traversability of AGV. They presented two approaches. One deals with the stereo data to classify drivable ground and uses the self-learning method. The other one uses a radar-stereo integrated system to detect and classify obstacles. They have done field experiments in different environments, including rural areas, agricultural land, etc.

A low-cost and multi-sensor-based obstacle detection method has been developed by Giannì et al. [46]. They have unified three sensing technologies: radar, lidar, and sonar. Then, the data is “sieved” and passed to the Kalman filter, and this technique estimates the distance of the obstacle accurately. Meichen et al. [47] take a similar Kalman filter-based approach. They have selected IMU (Inertial Measurement Unit) and lidar sensors to collect the coordinates of the obstacle, and both coordinates are fused to get the obstacle position.

Kragh and Underwood [48] used semantic segmentation by fusing lidar and camera sensors. The appearance information comes from a 2D camera, and the geometry information comes from 3D lidar data, and both information has been fused to get the final detection result. The advantage of this model is that it can differentiate between traversable overgrown grass or fallen leaves and non-traversable trees and bushes. Furthermore, using this method, ground, sky, vegetation, and object can be classifiable.

Ollis and Jochem [49] used a set of sensors to generate a “density map” to detect different obstacles and classify terrain. They used various ladar and radar sensors that can update 70 times in a second. The terrain has been divided into six subclasses based on the obstacle. They also classified traversable regions based on the difficulty level, such as non-traversable, traversable, partially traversable, etc.

Bradley et al. [50] utilized the infrared ray for detecting vegetation that has previously been used to detect chlorophyll. A near-infrared and a video camera are fused to capture image data. The visible light has been removed from each pixel of the image by applying a threshold value. Thus, the presence of chlorophyll or vegetation can be known.

A different approach has been adopted by Nguyen et al. [51]. They have not used any visual method; instead, they used a motion compensator and motion detector by blowing objects. They identified short or long grass and leaf branches in the front. However, this method is restricted to identifying and weighing passable vegetation when the vehicle is stopped.

For positive obstacle detection, researchers have fused different sensors in different ways. Lidar and cameras are mostly used sensors, and neural networks are commonly used algorithms. However, the Kalman filter, motion detector, and visual analysis are some methods that have delivered a reasonable detection result. In Table 3, we have summarized all the methods presented in Section 4, including the sensor, method, and obstacles that have been detected. Some works covered drivable ground or negative obstacles, along with positive obstacle detection.

## 5. Negative Obstacles Detection and Analysis

Positive obstacles are those objects with a positive height above the ground, while negative obstacles are those with a negative height below the ground [4]. Positive obstacles are easily visible and captured by the sensors. Because of the negative height and position below the ground, negative obstacles are somewhat challenging. Furthermore, a regular vision-based system may not measure their depth and area. It could be unsafe for the vehicle if the negative obstacles are not identified correctly. 

### 5.1. Missing Data Analysis

A common practice in negative obstacle detection is dealing with missing data from sensor signals. Many pieces of literature have worked with three-dimensional Lidar data. For example, Larson and Trivedi [52] have presented an algorithm for negative obstacle detection. They used a 3D laser to collect point cloud data. Two algorithms have been used for the classification method—Negative Obstacle DetectoR (NODR) and support vector machine (SVM). NODR, a geometry-based classifier, works by identifying missing information, which can lead to a negative obstacle. On the other SVM classifies the rays that return from Lidar. 

Sinha and Papadakis [53] also considered the information gap from 2D morphological images for negative obstacle detection. The advantage of their approach is that they process in real-time and provide a high-level accuracy without analyzing the 3D scene. The difference from other works is that they denoised the signals, extracted features through principal component analysis (PCA), and classified them according to the area.

Similarly, Heckman et al. [54] have also considered missing data from a 3D laser. By identifying areas where data is missing, they aim to detect the negative obstacles which could be a reason for missing data. The benefit of this method is that it can be applied to a complex environment even with sloped ground.

Analyzing missing data from the sensors has been a unique but effective method for detecting negative obstacles. The data come from the point cloud, lidar, or laser sensor for missing data analysis.

### 5.2. Vision-Based Detection

Much literature focuses on stereo vision to detect negative obstacles like the Lidar sensor. Karunasekera et al. [55] utilized the stereo camera’s 3D and color information. They generated a disparity map, where v-disparity and u-disparity have been applied to identify road profiles. U-V-disparity is an algorithm for understanding a 3D road scene, where it can classify different features of that scene [56]. Negative obstacles can be identified by scanning through every pixel of the disparity map.

Shang et al. [4] also used Lidar to collect data about negative obstacles. They mounted the sensor in an upright position in their work, which has a great advantage. The vehicle collects more information about its blind spot and data in this position. The width and the background information have been fused with the Bayesian network. Then, they used SVM classifiers to get the final detection.

Bajracharya et al. [57] used stereo vision for a special vehicle to detect sparse vegetation and negative obstacles. So, they build a terrain map and walk along with it. The system uses spatial and temporal information, making detection results more accurate. This system can work in dynamic locations and weather, like rain, snow, and even at nighttime.

Hu et al. [58] fuse different geometric cues from stereo cameras in the image sequence to detect negative obstacles. The stereo images contain range, color, and geometric information, and a Bayesian network calculates the probability of detection. The benefit of this method is that the obstacles can be detected from a far distance.

In [59], the performance of seven obstacle identification methods with 21 obstacles has been examined. Among these, two of them are negative obstacles. One was detected with a stereo image, and another was on the local map through software.

Overall, negative obstacle detection using vision is a difficult task, which has been possible with some image processing techniques and stereo vision.

### 5.3. Other Methods

Dima et al. [33] presented a data fusion technique for various obstacle detection. They fused information from multiple laser finder sensors and multiple machine learning classifiers. Different obstacles have different features, and different classifiers are suitable for them. Therefore, they fused multiple information along with the classifiers, providing much better results. This approach provided good detection accuracy for thin and negative obstacles. 

The approach mentioned in [43] is also applicable to detect negative obstacles. As this approach used the histogram elevation information to find the traversable region, the negative elevation objects are identified as negative obstacles.

The negative obstacle detection at nighttime has been addressed by Rankin et al. [60]. This work combined geometry information from stereo and thermal information from infrared. The thermal property is considered, and they considered that the interior of negative obstacles remains warmer than its surroundings throughout the night.

Goodin et al. [61] presented a Lidar-based model for analyzing the performance of negative obstacle detection. In this model, the sensor has to be installed on AGV to consider vehicle movement and speed, as the detection algorithm is based on curvature. This model has been cross-validated on Mississippi State University Autonomous Vehicular Simulator (MAVS) [62,63].

Morton and Olson [64] presented a mechanism considering three features, height, length, and density (HLD), for detecting both positive and negative obstacles. Height is such a parameter that distinguishes positive and negative obstacles. The main contribution of this work is that it can provide high accuracy with incomplete and noisy data.

Wang et al. [65] presented an obstacle detection method using a unique sensor, interferometric synthetic aperture radar (InSAR). InSAR has the ability to capture both scattering images and coherence images. In the detection approach, the shadow and edge information has been fused. The system differentiates the positive and negative obstacles by coherence area and amplitude.

As negative obstacles are somewhat difficult to detect, different other approaches have been taken to detect them. For example, color, height, thermal property, and curvature analysis. In Table 4, we have summarized all the methods presented in Section 5, including the sensors used, the methods, and the information about what they detected.

## 6. Discussion

In this paper, we have discussed different detection approaches, including drivable roads and obstacles, in off-road scenarios. The study of detection analysis is essential to ensure safety, smooth driving, and path planning [63] in an unknown environment. After reviewing the papers on different detection elements, some common criteria have been found. This section will explore and describe some key findings, challenges, and future directions from the overall review of the works presented in the previous sections.

### 6.1. Key Findings

#### 6.1.1. Sensors

Most works presented here are entirely or mostly dependent on one or more sensory information. The sensor provides the necessary information from the environment to a system; thus, the system learns about the environment from the data sensor provides and acts accordingly. Several works of detection rely on images, like monocular cameras, RGB cameras, or infrared cameras. Image-based sensors detect paths or obstacles based on visibility. Some detection works rely on the 3D point cloud system, like 3D lidar or laser sensors. In many off-road situations, 3D sensors have proven to be more dependable than 2D sensors for detection because the 3D sensor provides a clear picture of the surroundings, from where the system gets the idea of an object’s size, shape, and height. Furthermore, they are widely available at affordable prices.

Table 5 shows some of the sensors that are being used for detection, mostly on AVG, and the types of data they generate. Data resolution is also important for detection as it provides more detailed information about the objects/obstacles. Different types of sensors can be utilized depending on the vehicle and environment.

#### 6.1.2. Sensor Fusion

The environment is continuously changing through weather, dust, and daylight condition. Thus, the scene captured by a sensor change is not static. The variations in environmental parameters exacerbate sensory data and sensing performance. Therefore, one sensor may sometimes not be enough to adjust for these variations. Sensor fusion facilitates the creation of a reliable model that can accurately sense the environment under diverse environmental circumstances [68]. In addition, each sensor has its strength and weakness. Multiple sensors can overcome this weakness by filling a single sensor’s information gap and thus increasing detection accuracy. For example, obscured obstacles are challenging to detect with visually dependent sensors. Hollinger et al. [69] solved this difficult task by fusing lidar and radar. Both these sensors are based on radiofrequency. They tested this method with four different obstacles by placing them concealed by vegetation. The camera lidar fusion has been welcomed in much research, as a 2D camera alone cannot provide 3D information about a scene, so an additional lidar sensor might be required. The Kalman Filter and Bayesian reasoning are utilized to fuse the information from multiple sensors. Fusion can be performed at different levels, like low, mid, and high. For object detection, high-level information fusion may be required [70].

#### 6.1.3. Learning-Based Model

Many works of literature offer detection based on learning algorithms. The learning-based algorithms offer the system to have an understanding of the environment and allow it to get a better classification. As the system gets new data, it updates its logic automatically. The two primary learning-based methods are supervised and unsupervised learning. The training data are labeled in the supervised learning method, which is further used for testing or detection. In unsupervised learning, data are not labeled; but the system can label the data automatically with human involvement. The common part of these two algorithms is that they can predict new input. Another type of learning-based system is reinforcement learning, where the system learns from previous experiences by exploring the environment. Apart from classical machine learning, researchers have accepted deep learning, enabling a robust framework by adding more layers. However, increasing more layers to the network may also increase the complexity and computational time [71]. The popular learning-based model used in the literature for detection is SVM, CNN, deep neural network, etc. Some works developed their customized learning algorithms

### 6.2. Challenges

#### 6.2.1. Availability of Dataset

Autonomous technology has become an emerging technology. Researchers are continuously publishing new datasets to enrich this field day by day. A large group of researchers has devoted themselves to collecting and creating new datasets, but a huge portion of them is only limited to on-road driving. Furthermore, it is easier to get data from an urban environment instead of a rural atmosphere. Furthermore, only having the data may not be enough; annotating is really important. There are many data labeling tools available, which are mostly applicable to a structured environment. Therefore, many datasets are readily available, which are also labeled [72,73,74]. However, there are very few labeled datasets available in the off-road environment. Because of the lack of data availability, research in this field is very limited. Moreover, labeling off-road data is difficult because data labeling tools like ImageTagger [75] and OneLabeler [76] are sometimes challenging for unknown objects. Table 6 presents some popular publicly available datasets for autonomous vehicles. As we can see, many datasets are available for urban environments; off-road environments have fewer datasets.

#### 6.2.2. Hanging Obstacles Detection

Besides positive and negative obstacles, different types of obstacles can also be identified. They are hanging or dangling obstacles. Some good examples of hanging obstacles found in off-road environments are tree branches, rope, moss, etc. It is very important to identify such obstacles; otherwise, they can disrupt sensor vision and potentially damage vehicles. It is often mixed with positive obstacles, but they are not similar to other obstacles. A typical presumption is that an obstacle would always be on the same ground plane. However, hanging obstacles have been positioned some level above the ground. Though it is an important parameter, almost any work has been focused exclusively on hanging obstacle detection. Only Liu et al. [19] mentioned hanging obstacles that can be detected with other parameters using their technique. Some other works can be found to detect hanging obstacles for blind people using ultrasonic sensors [91]. Likewise, hanging obstacle detection for the industrial vehicle has been presented in [92]. Though their method is intended for indoor environments, it can experiment if those apply to outdoor off-road scenarios.

#### 6.2.3. Sensor Issues

A well-performed sensor system is very important for any detection. A suitable sensor is associated with cost, configuration, alignment, and sensor driver. Furthermore, mounting a sensor into the vehicle is a challenging task in maintaining all the requirements of the manufacturer. Many complexities may arise regarding inadequate sensor mounting. For example, while collecting data from the sensors, the alignment should be correct so that they can provide appropriate information to the system. Taking data from an incorrect position or an incorrect distance may deliver improper readings. As a result, it may not provide the right detection result, which may further cause false detection. In state-of-the-art, most of the sensor capabilities have been measured by the detection accuracy without considering incorrect measurements. Apart from the sensor alignment or the reading error, there are some other reasons for which a false detection may occur. For example, reflection from an object can mislead the sensor detection capability. A work presented by Peasley and Birchfield [66] considered this issue, and so they proposed a projection scheme and control strategy to solve this problem. Their techniques can work well in any uncertain environment.

#### 6.2.4. Environmental Challenges

As the environment is dynamic and its parameter continuously changes. Therefore, sensors sometimes fail to cope with this transformation. The environmental challenge is an important factor both in an on-road and off-road environment. However, environmental factors make the off-road vehicle more challenging. For instance, sometimes, the drivable path becomes very hard to detect because the road may not be visible in case of heavy rain or snow. In the on-road scenario, the vehicle may get information based on the structured environment or road signs even if the road marks are not visible, which is not possible in the off-road environment. Limited lighting conditions or nighttime becomes a problem when the system depends on only a vision or camera-based system. If the camera cannot capture clear images adequately, detecting objects and moving forward might be challenging. In addition, moving objects due to the high wind or waving trees/leaves need to be considered while driving because it would be challenging to learn about a moving object’s shape, size, and height.

#### 6.2.5. Real-Time Detection

In order to achieve a robust detection system, only sensor effectiveness and ability may not be enough. Getting the detection result at a perfect time is very crucial. The real-time analysis allows the system to react immediately. A slow reaction time may cause a collision, collapse, or damage. AGV detection systems must be real-time if we want vehicles to move fast and smoothly. So, the computational time and system memory should be at a considerable level. The majority of works primarily emphasize detection accuracy and not the processing time. The reason is that if the number of input samples grows, the computational cost also grows. Therefore, the method struggles to meet real-time restrictions. Furthermore, some data like stereo or RGB images have large sizes, which takes a long calculation time, and requires powerful GPUs. There is no doubt about the importance of detection accuracy but getting the detection result at the right time helps to make decisions at the right time. An algorithm SVR developed in [42] can reasonably reduce the computational time. There are many real-time object detection algorithms available for the structured environment, like YOLO (You Only Look Once) [93], deep CNN [94,95], etc. Few attempts have been made to try these algorithms in off-road datasets.

### 6.3. Future Possibilities

In the last part, we have mentioned the major challenges of the existing detection works in unstructured environments. The future target could be overcoming or minimizing these challenges. For example, publish more off-road data to society so that more experiments can be done in this field. Hanging obstacle detection could be a potential area of interest, as any work in this sector has rarely been done. Sensors mounting in different positions may impact capturing data and thus the detection.

As we have seen, sensor fusion has proven to be a useful method. However, more sensor fusion can be a complex problem and not cost-effective. Finding an optimal solution is essential for this case. Training different machine learning models is also challenging due to the lack of labeled data. The recent development of semi-supervised learning [96] or active learning [97] has become popular as labeling data is time-consuming and difficult. Existing algorithms that work for an on-road environment may not be directly used in off-road scenarios. However, existing algorithms can be improved or apply transfer learning to off-road environments. Using a complex algorithm may have been valuable, but a complex algorithm requires a lot of processing time, and having a computational cost may not fulfill the real-time analysis requirement. So, research on how to reduce computational complexity would be beneficial to autonomous society.

As we mentioned, sensor fusion and advanced machine learning algorithms can help overcome driving difficulties in off-road environments. Some popular deep learning networks for object detection are YOLO [93], R-CNN [98], SSD (Single Shot multi-box Detector) [99], Mobilenet [100], SqueezeDet [101], etc. Besides these networks, CNN-based 3D object detection is very popular for autonomous driving [102,103,104]. Those techniques have been applied in on-road circumstances, which should also be applied in an unstructured environment. Artificial intelligence (AI) plays a significant role in perception, path planning, and control techniques in various complex environments. Yet, there is much scope to improve the performance of AGV [105]. However, implementing vehicular algorithms requires high computing to meet this demand. Few works have addressed the issue of communicating vehicles with different domains [106,107]. These methods can be tested in off-road environments.

Introducing more intelligence and using localization sensors such as Global Navigation Satellite Systems (GNSS) [108] or global positioning systems (GPS), AGVs get information about their positions and then can localize themselves in known and unknown environments. As wireless physical layer technologies can generally adapt to the wireless environment, their combination with reconfigurable surfaces and deep learning approaches can open up new paths to secure 6G vehicular-aided heterogeneous networks [109,110]. Vehicular edge computing can reduce computational time via optimal computational and communication resource allocation [111].

Real-time processing for sensor data has become challenging in the automotive industry as it requires more computational power and time. Computationally intelligent and energy-efficient data sharing among various onboard sensors need an advanced optimization framework to minimize the total transmit power of the vehicle-to-everything (V2X) networks [112]. Besides, sensor data can be corrupted by different noise models [113], so the noisy data need to be removed for better detection [114]. YOLO with adaptive frame control has been used for real-time object detection in AI-embedded systems [115]. In the case of navigation, the active learning algorithm [116] shows good potential for low infrastructure and the off-road environment for automated driving [117].

This work has not included environment detection in any entirely unknown environment as it is out of scope for this work. However, environment detection is important for planning a framework for the trajectory of AGV. Some research works are going on to address environment detection [118]. In the future, this topic would be a great addition to the off-road environment study for autonomous vehicles.

## 7. Conclusions

Detection is a key capability of AGV. This work provides a detailed overview of the detections of AGV in off-road environments. The off-road environment has some limitations over the on-road; therefore, detection in an off-road environment is more challenging. For detection, we classified some significant features for unstructured settings. The drivable ground and the obstacles are two primary components that need to be detected for safe navigation. Obstacles themselves can be divided into multiple categories. In this study, positive and negative obstacle detection has been studied. We found no paper in the literature solely based on hanging obstacle detection for off-road environments.

Most of those detection techniques mainly depend on different sensing technologies and learning algorithms. Lidar, camera, radar, infrared, laser, and stereo are commonly used for detection procedures. CNN, supervised learning, unsupervised learning, SVM, and deep learning are some frequently used algorithms. There are many advancements have been made in detection techniques. However, we highlighted some challenges that still need to be solved. For example, the lack of available data, sensor alignment and false detection, the complexity of real-time analysis, environmental difficulties, etc., must be addressed.

There are many scopes to improve detection performance, using sensor fusion, AI, remote sensing, applying new algorithms, and reducing computational complexity. Furthermore, real-time analysis, 3D object detection, and V2X connectivity have good potential. We look forward to more emphasis on overcoming these challenges by the researchers in the upcoming days. Furthermore, we believe this work will help the reader in finding literature who are doing similar works.

## Figures and Tables

**Table 1:** The existing review works that have covered the mobility of AGV in off-road environments, including this work.

Literature	Published Year	Area Covered	Technology Focused
Chhaniyara et al. [12]	2012	Terrain trafficability analysis	Remote sensing technology
Papadakis [13]	2013	Terrain traversability analysis	Sensor technology
Ilas [14]	2013	Electronic sensing technologies	Sensor technology
Babak et al. [15]	2017	The advancements in AGV technology	Sensor technology
Lynch et al. [16]	2019	Sensor technologies	Sensor technology
Hu et al. [17]	2020	Sensor fusion-based obstacle detection	Sensor technology
Guastella et al. [18]	2021	Environmental perception	Learning-based methods
This article	2022	Ground, positive, and negative obstacles detection	Both sensor technologies and learning-based methods

**Table 2:** The methods used for traversable ground detection.

Literature	Sensors	Method	Detection
Gao et al. [20], 2019	Lidar	Deep Learning	Drivable ground
Chen et al. [21], 2017	Lidar	Lidar-histogram	Obstacles and drivable ground
Liu et al. [19], 2019 Zhu et al. [25], 2019		Radial and Transverse feature Bayesian Network	
Katramados et al. [22], 2009	Camera	Vision/Traversability map	Drivable ground
Shaban et al. [23], 2021	Lidar Camera	Semantic Segmentation	Drivable ground
Gao et al. [24], 2021 Dabbiru et al. [9], 2021			
Tang et al. [28], 2018	Laser + Camera	Unsupervised Learning	Drivable ground
Reina et al. [30], 2016 Dahlkamp et al. [26], 2006	Lidar/Laser + Camera	Supervised Learning/SVM	Drivable ground
Sock et al. [31], 2016 McDaniel et al. [32], 2012			

Support Vector Machine (SVM).

**Table 3:** The methods used for positive obstacle detection.

Literature	Sensors	Method	Detection
Huertas et al. [34], 2005, Broggi et al. [38], 2005	Camera	Stereo Algorithm V-disparity	Positive obstacles/vegetation
Maturana et al. [35], 2018, Foroutan et al. [40], 2021	Lidar/Camera	CNN/Machine learning	Positive obstacles
Manderson et al. [36], 2020, Nadav et al. [37], 2017	Camera	Supervised Learning	Positive obstacles
Kuthirummal et al. [43], 2011	Lidar/Camera	Graph Traversal Algorithm	Positive + Negative obstacles
Chen et al. [41], 2020	Laser	(LM-BP) neural network	Positive obstacles
Zhang et al. [42], 2014	Camera	Stereo vision + SVR	Positive obstacles
Bradley et al. [50], 2004	Infrared + Camera	Vision	Positive obstacles/vegetation
Reina et al. [45], 2017, Manduchi et al. [44], 2005	Camera + Ladar/Radar	Supervised Learning	Positive obstacles
Giannì et al. [46], 2017	Radar+Lidar + Sonar	Kalman filter	Positive obstacles
Ollis and Jochem [49], 2013	Radar + Ladar	Density Map	Positive obstacles + drivable ground
Kragh and Underwood [48], 2020	Camera + Lidar	Deep learning	
Nguyen et al. [51], 2012	Blowing object	Motion compensation and detection	Positive obstacles/vegetation

Convolutional Neural Network (CNN); Levenberg–Marquardt back-propagation (LM-BP); Space-Variant Resolution (SVR).

**Table 4:** The methods used for negative obstacle detection.

Literature	Sensors	Method	Detection
Larson and Trivedi [52], 2011 Sinha et al. [53], 2013 Heckman et al. [54], 2007	Lidar/Laser	Missing data analysis	Negative obstacles
Shang et al. [4], 2014	Lidar	SVM	Negative obstacles
Rankin et al. [60], 2007	Infrared	Thermal property analysis	Negative obstacles
Rankin et al. [59], 2005 Hu et al. [58], 2011 Bajracharya et al. [57], 2013 Karunasekera et al. [55], 2017	Camera	Vision	Negative obstacles
Goodin et al. [61], 2021	Lidar	Curvature analysis	Negative obstacles
Dima et al. [33], 2004	Camera+ Lidar	HLD	Positive + Negative obstacles
Morton and Olson [64], 2011	Camera+ Infrared camera+ Laser	Color and texture analysis	
Wang et al. [65], 2016	InSAR	Data Fusion	

Negative Obstacle DetectoR (NODR); Principal Component Analysis (PCA); Height, Length, and Density (HLD).

**Table 5:** The sensors usually used for detection.

Sensors	Data Type	Resolution
Camera [66]	Images	High
Lidar [46]	Point clouds	High
Radar [46]	Radio frequency	Low
Laser [66]	Signal reflectivity	High
Ladar [67]	Signal reflectivity	High
Infrared [60]	Electromagnetic radiation	Low
Stereo [42]	Radio frequency	High
Sonar [46,66]	Sound reflection	Low

**Table 6:** Some publicly available datasets for autonomous vehicles.

Dataset Name	Purpose	Data Type	Application
Astyx Dataset HiRes2019 [77]	3D Object detection	Radar-centric information	On-road
Berkeley DeepDrive [78]	Obstacles, drivable areas, and lane detection	Video sequence	On-road
Landmarks [79]	Landmark detection	Camera images	On-road and Off-road
Level 5 [80]	Traffic agent and path detection	Camera and lidar images	On-road
nuScenes Dataset [81]	Object detection	Camera and lidar images	On-road
Open Images V5 [82]	Object detection	Camera images	On-road
Oxford Radar RobotCar [83]	Path planning	Radar route	On-road
Pandaset [84]	Understanding scenarios	Camera and lidar images	On-road
Waymo Open Dataset [85]	Understanding scenarios	Video sequence	On-road and Off-road
RELLIS-3D Dataset [86]	Object detection	Camera and lidar images	Off-road
CaT: CAVS Traversability Dataset [87]	Traversability	Camera images	Off-road
Off-road Terrain Dataset [88]	Understanding scenarios	Camera images	Off-road
Freiburg Forest [89]	Understanding scenarios	Camera images	On-road and Off-road
ROOAD [90]	Localization	Camera images	Off-road

RELLIS Off-road Odometry Analysis Dataset (ROOAD).

## Abbreviations

The following abbreviations are used in this manuscript:

AI	Artificial Intelligence
AGV	Autonomous Ground Vehicle
ALV	Autonomous Land Vehicles
BVNet	Bird’s Eye View Network
CaT	CAVS Traversability Dataset
CNN	Convolutional Neural Network
CAVS	Center for Advanced Vehicular Systems
DARPA	Defense Advanced Research Projects Agency
GNSS	Global Navigation Satellite Systems
GPS	Global Positioning Systems
HLD	Height, Length, and Density
IMU	Inertial Measurement Unit
InSAR	Interferometric Synthetic Aperture Radar
LADAR	Light and Radio Detection and Ranging
Laser	Light amplification by stimulated emission of radiation
LIDAR	Light Detection and Ranging
LM-BP	Levenberg–Marquardt back-propagation
MAVS	Mississippi State University Autonomous Vehicular Simulator
MLP	Multilayer Perceptron
NODR	Negative Obstacle DetectoR
PCA	Principal Component Analysis
SONAR	Sound Navigation and Ranging
SSD	Single Shot multi-box Detector
SVM	Support Vector Machine
SVR	Space-Variant Resolution
RADAR	Radio Detection and Ranging
ROOAD	RELLIS Off-road Odometry Analysis Dataset
R-CNN	Region-based Convolutional Neural Network
TTA	Terrain Traversability Analysis
UGV	Unmanned Ground Vehicle
YOLO	You Only Look Once
